# Comparing sea ice habitat fragmentation metrics using integrated step selection analysis

**DOI:** 10.1002/ece3.6233

**Published:** 2020-04-12

**Authors:** Brooke A. Biddlecombe, Erin M. Bayne, Nicholas J. Lunn, David McGeachy, Andrew E. Derocher

**Affiliations:** ^1^ Department of Biological Sciences University of Alberta Edmonton Alberta Canada; ^2^ Wildlife Research Division, Science and Technology Branch Environment and Climate Change Canada Edmonton AB Canada

**Keywords:** habitat fragmentation, integrated step selection analysis, polar bear, sea ice, spatial autocorrelation

## Abstract

Habitat fragmentation occurs when continuous habitat gets broken up as a result of ecosystem change. While commonly studied in terrestrial ecosystems, Arctic sea ice ecosystems also experience fragmentation, but are rarely studied in this context. Most fragmentation analyses are conducted using patch‐based metrics, which are potentially less suitable for sea ice that has gradual changes between sea ice cover, than distinct “long‐term” patches. Using an integrated step selection analysis, we compared the descriptive power of a patch‐based metric to a more novel metric, the variation in local spatial autocorrelation over time. We used satellite telemetry data from 39 adult female polar bears (*Ursus maritimus*) in Hudson Bay to examine their sea ice habitat using Advanced Microwave Scanning Radiometer 2 data during sea ice breakup in May through July from 2013–2018. Spatial autocorrelation resulted in better model fits across 64% of individuals, although both metrics were more effective in describing movement patterns than habitat selection. Variation in local spatial autocorrelation allows for the visualization of sea ice habitat at complex spatial and temporal scales, condensing a targeted time period of habitat that would otherwise have to be analyzed daily.

## Graphical abstract

Sea ice fragmentation does not create distinct patches in the same way terrestrial fragmentation does. We compared traditional patch‐based metrics to our novel spatial autocorrelation metric using integrated step selection analyses to determine which metric was better for predicting polar bear movement and habitat selection. Spatial autocorrelation provided a better model fit for 64% of individuals.

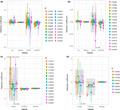

## 1 INTRODUCTION

Habitat fragmentation is the separation of continuous habitat into smaller, isolated patches, which often occurs with habitat loss (Fahrig, 2003; Wilcove, McLellan, & Dobson, 1986). As the amount of available habitat decreases, the distance between patches often increases resulting in greater fragmentation (Andren, 1994; Goodsell & Connell, 2002). The characteristics of the matrix between patches affect how fragmentation influences wildlife, based mainly on how organisms move among patches (Boudjemadi, Lecomte, & Clobert, 1999; Sondgerath & Schroder, 2002; Taylor, Fahrig, Henein, & Merriam, 1993).

For ice‐associated species, variation in sea ice cover (i.e., habitat) and open water (i.e., matrix) are comparable to patches in fragmented terrestrial landscapes (Sahanatien & Derocher, 2012). Arctic sea ice is a dynamic habitat in both space and time, which is affected by ocean currents, winds, and temperature (Markham, 1986; Saucier et al., 2004; Stern & Laidre, 2016; Wang, Mysak, & Ingram, 1994). Sea ice undergoes annual cycles, concurrent with the seasons, where ice thaws during breakup and southern Arctic regions become ice‐free in summer. Freeze‐up occurs in winter, and sea ice reaches its maximum extent in March (Stern & Laidre, 2016). Breakup reflects a period where sea ice declines rapidly and habitat continually changes until it is lost (Saucier et al., 2004; Stroeve & Notz, 2018). Although the timing follows a predictable annual pattern, the rapid changes means the length, start, and end of breakup are variable (Gagnon & Gough, 2005). Sea ice change is variable across the Arctic, but over the last four decades, all regions have had an increased ice‐free season and a decrease in ice cover when present (Comiso, 2012; Parkinson, 2014; Stern & Laidre, 2016). Temporal variation in sea ice cover adds complexity to spatial variation, affecting the species that use this habitat. The importance of sea ice has been addressed for multiple ice‐associated species, but sea ice fragmentation has rarely been explored in the same context.

Many species in Arctic marine ecosystems are partially or wholly ice‐associated. The presence, absence, and variability of sea ice affect the space use of organisms that live in, on, and underneath the ice (Arndt & Swadling, 2006; Mallory, Gaston, Gilchrist, Robertson, & Braune, 2010; Post et al., 2013; Wassmann, Duarte, Agusti, & Sejr, 2011). Marine mammals exhibit multiple uses for sea ice, including habitat for mating, giving birth, raising offspring, molting, resting, and foraging on ice‐associated prey (Kovacs, Lydersen, Overland, & Moore, 2011; Laidre et al., 2008; Wassmann et al., 2011). The location of sea ice is often important, as it allows many species to haul out over shallow water to remain close to high productivity regions for foraging (Bluhm & Gradinger, 2008; Kovacs et al., 2011; Wilson, Regehr, Rode, & St Martin, 2016). Polar bears (*Ursus maritimus*) are strongly associated with ice fragmentation as they only stay in the marine environment when sea ice is present (Cherry, Derocher, Thiemann, & Lunn, 2013; Ferguson, Taylor, Born, Rosing‐Asvid, & Messier, 2001; Stirling, Andriashek, & Calvert, 1993; Stirling, Jonkel, Smith, Robertson, & Cross, 1977). They are highly dependent on sea ice as a substrate from which they access prey, travel, and mate (Smith, 1980; Thiemann, Iverson, & Stirling, 2008); therefore, changes in its presence, structure, or fragmentation reduces available habitat quality (DeMaster & Stirling, 1981; Galicia, Thiemann, Dyck, Ferguson, & Higdon, 2016; Kingsley, Stirling, & Calvert, 1985; McCall, Pilfold, Derocher, & Lunn, 2016; Sahanatien & Derocher, 2012; Stirling & Archibald, 1977). Polar bear habitat use varies seasonally, where bears select for high ice cover when it is available and more open ice during seasons when ice cover is lower (Mauritzen et al., 2003; McCall et al., 2016; Wilson et al., 2016). Breakup reflects a period where sea ice cover rapidly declines and polar bears exhibit individual variation in habitat use that reflects those changes (McCall et al., 2016). Quantifying changes in fragmentation during breakup may be crucial for understanding hunting success, energetics, migration, and the initiation of on land fasting period when bears can no longer access seals (Cherry et al., 2013; Sahanatien & Derocher, 2012; Watts & Hansen, 1987).

Habitat fragmentation is often analyzed with the goal of determining characteristics such as extent of isolation, connectivity, or amount of viable habitat (Fahrig, 2003). Fragmentation analyses are often conducted using patch‐based metrics, such as patch size, distance between patches, and patch shape (i.e., Bender, Tischendorf, & Fahrig, 2003; Liu, Zhang, Zhang, Musyimi, & Jiang, 2014; Petrasova‐Sibikova, Bacigal, & Jarolimek, 2017; Sahanatien & Derocher, 2012). Patch‐based metrics have been used in many ecosystems and across scales to classify landscape patterns and to evaluate how landscapes change over time (Haddad et al., 2015). In most ecosystems, monitoring annual or even decadal changes in various aspects of fragmentation are often sufficient to understand how wildlife will react to fragmentation (Echeverría et al., 2006; Keleş, Sivrikaya, Çakir, & Köse, 2008; Reddy, Jha, & Dadhwal, 2013). However, in Arctic marine ecosystems, patch‐based metrics may miss fine‐scale spatial variation of fragmentation that occurs on daily time scales. Despite parallels with fragmentation of terrestrial systems, use of patch‐based metrics to understand wildlife behavior in Arctic marine systems is uncommon. This is in part because gradual changes between sea ice cover and the temporal dynamics make the delineation of daily patches data intensive and thus, analytically challenging.

An alternative approach to quantify habitat fragmentation in dynamic landscapes, where patch edges are not particularly discrete, is spatial autocorrelation (Fan & Myint, 2014; Fan, Myint, & Zheng, 2015; Pearson, 2002; Roberts, Hall, & Calamai, 2000). Spatial autocorrelation can be used to quantify habitat continuity at a local scale, which describes variability in habitat cover across an entire habitat. In terms of sea ice, positive spatial autocorrelation reveals regions of similar percentage sea ice cover. Negative spatial autocorrelation reflects regions of discontinuous habitat, where sea ice cover is inconsistent. There is potential for spatial autocorrelation metrics to address the spatially dynamic component of sea ice habitat because of their ability to quantify fragmentation without the creation of patches. The spatial variation quantified by local spatial autocorrelation can be augmented to include temporal variation by considering the variation in spatial autocorrelation over time.

The objective of this study is to compare spatial autocorrelation and patch‐based metrics of habitat fragmentation to assess which method better describes polar bear habitat use and movement using integrated step selection analyses. We analyze polar bear telemetry data from adult female polar bears in Hudson Bay. We hypothesize that ice‐associated species like polar bears respond to fine‐scale variation in sea ice cover, and therefore we predict that the spatial autocorrelation metric will be the better descriptor of polar bear movement than patch‐based metrics.

## 2 METHODS

### 2.1 Study area

**Figure 1:**
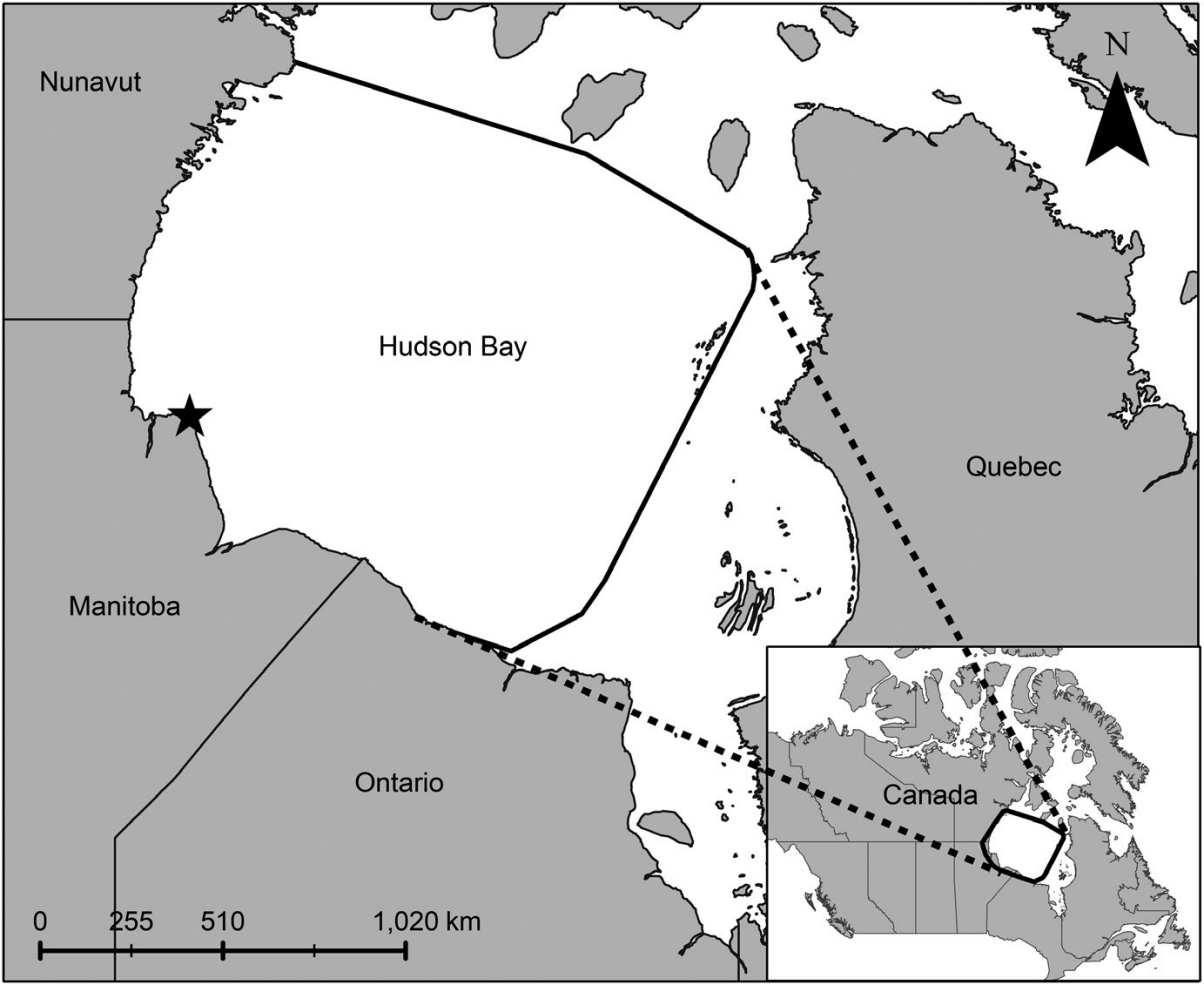
Map of the study area in Hudson Bay, trimmed to the coastline, which was determined with a 100% minimum convex polygon from satellite telemetry locations of adult female polar bears, 2012–2018 (*n* = 65). Cape Churchill, a proxy for polar bear summer refuge, is denoted by a star

Hudson Bay, Canada, (Figure 1) is a shallow (mean depth of <150 m) inland sea, covering 1,240,000 km^2^ (Macdonald & Kuzyk, 2011). The Bay undergoes an annual cryogenic cycle with general trends of sea ice present from November to June followed by an ice‐free period from July to October (Gough & Wolfe, 2001). Ice presence and absence are punctuated by periods of ice breakup in May–July and freeze‐up in November–December (Castro de la Guardia, Myers, Derocher, Lunn, & Terwisscha van Scheltinga, 2017). Our study focuses on the Western Hudson Bay (WH) polar bear population, which has declined by ~30% over the past four decades and has been linked to a decline in the length of ice presence (Lunn et al., 2016).

### 2.2 Animal data collection

Adult (≥5 years old) female polar bears were immobilized via remote injection of Zoletil® (Virbac S.A., Carros, France) following standard protocols (Stirling, Spencer, & Andriashek, 1989) from 2012 to 2017 as part of ongoing, long‐term research on the ecology of the WH population (i.e., Derocher & Stirling, 1995; Lunn et al., 2016; Ramsay & Stirling, 1988; Regehr, Lunn, Amstrup, & Stirling, 2007; Stirling, Lunn, & Iacozza, 1999). Capture and handling protocols were reviewed and approved annually by the Environment and Climate Change Canada, Western and Northern Animal Care Committee and the University of Alberta Biological Sciences Animal Policy and Welfare Committee in accordance with the Canadian Council on Animal Care. Bears were fitted with Argos or Iridium satellite‐linked geographic position system collars (Telonics, Mesa, AZ, USA) that were programmed to record locations at 4‐hr intervals and equipped with a mechanism programmed to release after one or two years. Locations where rates of movement were biologically impossible were removed (i.e., >30 km/hr). The study area was delineated by a 100% minimum convex polygon of telemetry locations, trimmed to the Hudson Bay coastline to include only marine locations (Figure 1).

### 2.3 Quantifying fragmentation

Monthly ice coverage data were collected via the Advanced Microwave Scanning Radiometer 2 (AMSR‐2) on the GCOM‐W1 platform with a resolution of 6.25 km × 6.25 km grid cells from 2013 to 2018 (Spreen, Kaleschke, & Heygster, 2008). The data were obtained from the National Snow and Ice Data Center (Boulder, CO). This is the highest resolution data covering our study period and area. Each grid cell, or pixel, had a value from 0 to 200 defining ice cover, which was divided by 2, resulting in values from 0–100 to reflect percentage of sea ice coverage. Within each pixel, ice cover ≥60% was optimal habitat and <60% was considered suboptimal habitat based on other studies where preferred polar bear habitat was defined (Cherry, Derocher, & Lunn, 2016; Cherry et al., 2013; Laidre et al., 2015; Lone, Merkel, Lydersen, Kovacs, & Aars, 2018; Mauritzen et al., 2003; Pilfold, Derocher, & Richardson, 2014; Sahanatien & Derocher, 2012). We used ArcGIS version 10.5.1 (Environmental Systems Research Institute, 2017) to classify the daily ice cover as either optimal or suboptimal habitat. We computed global percentage of optimal habitat (PHAB: called PLAND in the software) using FRAGSTATS (McGarigal, Cushman, & Ene, 2012) to determine the daily amount of available habitat from 2013 to 2018. In this context, global refers to the entire study area. We examined the breakup period, defined by the first date in any year where PHAB dropped and remained below 95%, until the day before PHAB dropped and remained below 5%. We further separated the breakup period into early and late, defined by the date that PHAB reached 50% in each year.

To create our local habitat fragmentation metrics for comparison we used a moving window around each pixel to scale fragmentation to the local potential habitat an individual bear could experience in one day. We used the mean daily distance travelled by collared bears in the study during breakup (21 km/day, *SD* = 5.3, range 11–31 km/day) as a radius to define an area of potential daily use. This resulted in an area of 1,385 km^2^, which we translated into ~35 6.25 × 6.25 km pixels and rounded to a 25 pixel (5 pixel × 5 pixel) moving window, because local analyses required a central pixel.

To create a spatial autocorrelation metric to compare to patch‐based metrics we used local Geary's *c*, a local indicator of spatial association (Anselin, 1995; Haining, 1993). We calculated Geary's *c* during breakup across all years. We used Geary's *c* because it focuses on dissimilarity, where greater values reflect negative spatial autocorrelation (Anselin, 1995, 2018) and identify regions where sea ice cover is more variable and thus fragmented. Geary's *c* values can range from 0 to unspecified values >1. Values close to 0 reflect positive spatial autocorrelation and large values reflect negative spatial autocorrelation which describes fragmented, discontinuous sea ice. The statistic for each pixel was calculated using the 5 × 5 pixel moving window defined above. Pixels around the edge of study area that did not have sufficient surrounding pixels to fill the moving window were assigned null values.

To assess the temporal dynamics of fragmentation we used the variation in spatial autocorrelation at each pixel in space over time. In each year, the standard deviation of the Geary's *c* value, or spatial autocorrelation standard deviation (SASD), for each pixel in the study area across early breakup was calculated and the resulting values were plotted. SASD was calculated for late breakup, resulting in two SASD raster files per year. Pixels with high SASD reflect locations where sea ice cover is highly variable and fragmentation most dynamic.

The 5 × 5 pixel moving window was also used for analysis of two local patch‐based metrics, total edge (TE) and percent habitat (PHAB), using FRAGSTATS metrics TE and PLAND via the *landscapemetrics* package in R (R Core Team, 2018, *v1.1*, Hesselbarth, Sciaini, With, Wiegand, & Nowosad, 2019). We chose TE and PHAB because melting causes sea ice patches to be spatially correlated during breakup, which precludes the use of configuration metrics in FRAGSTATS (Sahanatien & Derocher, 2012). The two habitat patch types were defined as above, as optimal or suboptimal habitat. Optimal habitat was used as the patch type of interest in analyses, which resulted in separate daily raster files for local TE and local PHAB. High TE values reflect regions with more edge and thus more fragmented habitat, and lower TE reflects regions of constant habitat or nonhabitat. High PHAB values reflect regions where local percent optimal habitat is high.

### 2.4 Integrated step selection analysis

Integrated step selection analysis (iSSA) furthers habitat selection analysis by incorporating movement parameters and defining availability by the distribution of the used movement metrics (Avgar, Potts, Lewis, & Boyce, 2016). The environmental covariates in an iSSA quantify habitat selection; the interaction terms between environmental covariates (when extracted at the start of the step) and movement parameters, turning angle and step length, quantify the effect the environment has on movement. The ability for an iSSA to estimate movement and habitat selection simultaneously is an asset because in complex habitats such as sea ice there is likely an interplay between what individuals are selecting for and how they move.

We developed four separate iSSAs using the *amt* package in R (Signer, Fieberg, & Avgar, 2019) to determine if SASD (spatial autocorrelation metric) or PHAB and TE (patch‐based metrics) were better descriptors of habitat selection and movement patterns by polar bears. Within our analyses, steps are defined as the connections between consecutive individual locations, step length is the distance between consecutive locations, and turning angle is the change in directionality between consecutive steps. Analyses were separated into early and late breakup, resulting in SASD early, SASD late, PHAB/TE early, and PHAB/TE late models. Polar bear locations were resampled using consecutive bursts of ≥3 steps with a 15‐min tolerance around a 4‐hr fix rate, to account for missing location points. Each resulting step was matched to 10 random steps, which were randomly generated using a gamma distribution for step length, and a von Mises distribution for turning angle (Avgar et al., 2016). To verify that the resolution of our habitat data was fine enough to detect variation in habitat use across polar bear steps, we quantified the percentage of steps that started and ended in the same habitat pixel. We found that 23% of steps start and end in the same pixel and only 20% of individuals had >23% of their steps start and end in the same pixel, suggesting that the percentage of same pixel steps is driven by a relatively small portion of the sample.

For SASD models, candidate environmental covariates of daily sea ice cover (ice), and SASD for early and late breakup were extracted at the end of both used and random steps. We also included the square of daily ice cover to test for a nonlinear relationship. We included the distance from each point to Cape Churchill on the west coast of Hudson Bay at the end of each step as a proxy for summer refuge (refuge) as a candidate covariate (Figure 1). Cape Churchill was chosen as a proxy for summer refuge because WH polar bears exhibit high fidelity to refugia along this area of the coast (Derocher & Stirling, 1990; Stirling, Lunn, Iacozza, Elliott, & Obbard, 2004) but the jut of the coast can confound distance to coast measurements, thus a single point was chosen. We tested interaction terms: SASD with the natural log of step length, and SASD with the cosine of turning angle, where SASD was extracted at the start of each step. These interaction terms examined how individual movement was affected by changes in SASD. Analyses were conducted for each bear in early and late breakup separately.

Candidate environmental covariates for patch‐based models included daily sea ice cover (ice), PHAB, and TE, and were extracted at the end of used and random steps. Similar to above, the distance to summer refuge (refuge) was also a candidate covariate extracted at the end of each step. We also included PHAB and TE interacting with the natural log of step length, as well as PHAB and TE interacting with the cosine of turning angle, using PHAB and TE extracted at the start of each step to analyze movement. We examined all daily covariates for collinearity and excluded any with Pearson correlation values >|0.6| from the same models. Collinear covariates were tested in separate models and the one with better fit, determined by AIC, was retained. Individuals with all locations in regions of solely optimal or suboptimal habitat locally resulted in no variation in PHAB and were removed from further analysis.

In each of the four model groups, SASD early, SASD late, PHAB/TE early, and PHAB/TE late, all combinations of all corresponding candidate covariates and interactions outlined above were tested for each individual. Akaike information criteria (AIC) was used for each individual in each group to determine model of best fit, where the top‐ranked model was >2 AIC lower than the next ranked model. Polar bears are individually variable (McCall et al., 2016) thus we expected our chosen covariates to explain some individual's behavior better than others. AIC was expected to have some limitations in determining best model fit across multiple individuals, so we kept individuals separate and chose the top model based on majority. The most common top‐ranked SASD and PHAB/TE models across all individuals in early and late breakup were chosen to facilitate the comparison between SASD and PHAB/TE. For each individual, we compared the Cox & Snell pseudo‐R‐squared values of the top SASD and PHAB/TE iSSA models to determine which fragmentation metric had better model fits (Cox & Snell, 1989). Results are presented as beta coefficients for each covariate in the top models and 95% confidence intervals.

## 3 RESULTS

There were 39 collared polar bears in early breakup, and 29 of those maintained locations into late breakup. Maximum speed of bear movement was 15.5 km/hr.

The global percent of optimal sea ice habitat (PHAB) was similar across all years, with a significant sigmoidal trend in mean global PHAB during breakup (logistic regression: horizontal asymptote = 90.8%, *p* < .001; x‐value at half asymptote = June 24, *p* < .001; scale = −8.7, *p* < .001). Mean breakup period lasted 63 days (*SD* = 15.7, *n* = 6) and ranged from 44 days in 2014 to 81 days in 2015. The mean date for start of breakup was May 12 (range May 2 – May 29, *SD* = 10.0, *n* = 6). The mean date for end of breakup was July 14 (range July 6 – July 23, *SD* = 7.2, *n* = 6). The breakup period based on global PHAB was May 2 to July 23. The start of the second half of breakup was June 24, June 23, June 23, June 30, June 17, and June 26, in 2013–2018, respectively.

Late breakup in 2017 had the highest SASD, with a maximum pixel value in the study area of 1556, the next highest was late breakup in 2013, with a maximum SASD of 393 (Figure S1). The period with the lowest maximum SASD was late breakup in 2015, with a maximum SASD of 56. There were qualitative differences in sea ice cover (Figure S1). Low SASD occurred consistently across all years in early breakup, mostly notably in the South‐East, as this is where sea ice remains the longest and is the most intact. Conversely, the least variable regions in late breakup are open water regions, where the sea ice is lost earliest. The most variable regions in late breakup reflect regions where ice persists the longest and occurred in the south, along the south‐west coast.

**Figure 2:**
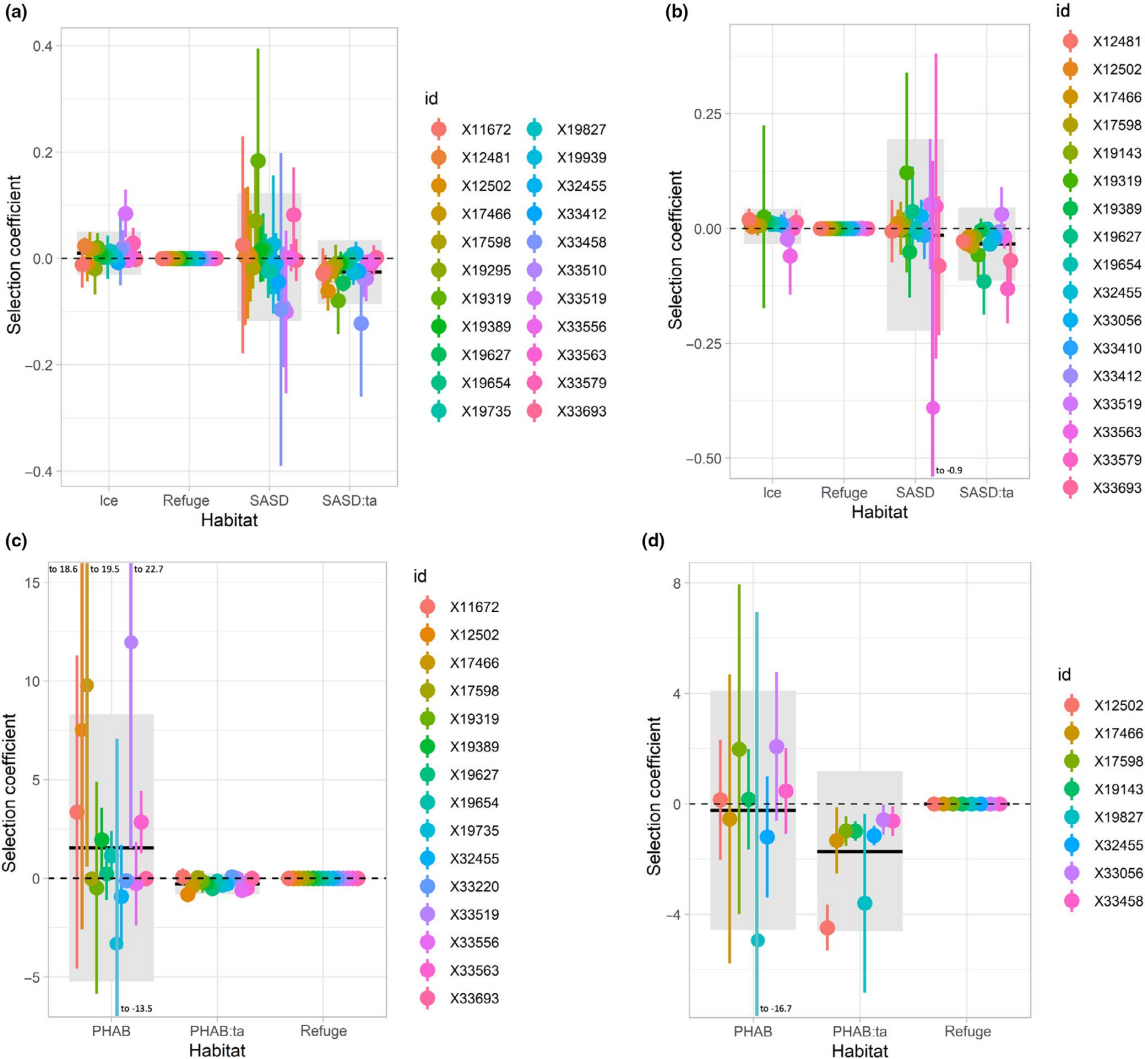
Integrated step selection analysis beta coefficients (points) and 95% confidence intervals (vertical lines) for individual adult female polar bears in Western Hudson Bay with ≥ 1 significant covariate in the top model for each model group: (a) SASD early (one individual with low SASD and SASD:ta coefficients was removed as an outlier), (b) SASD late, (c) PHAB early, and (d) PHAB late. Black bars show population mean beta coefficients for each covariate and gray boxes show 95% confidence intervals

For spatial autocorrelation analysis, the top iSSA model for the majority of individuals included the covariates ice, SASD, and refuge, as well as the interaction between SASD and the cosine of turning angle (Table 1). The AIC values for multiple individuals were indistinguishable across the top three models (raw AIC values in Table S1). For 23 of 39 individuals in early breakup, the top model had beta coefficients that significantly differed from zero in ≥1 covariate (Figure 2a). There were 11 individuals with a significantly negative interaction between SASD and the cosine of turning angle. The negative interaction term reflects increased SASD significantly related to increased changes in the direction of travel (i.e., more deflection from a straight path from step to step). In late breakup, 17 of 29 individuals had ≥1 significant covariate in the top model (Figure 2b). There were 13 individuals with a significantly negative interaction term, reflecting increased changes in the direction of travel with higher SASD.

**Table 1:** Number of individuals with the lowest AIC (by Δ2 or greater) for the top three spatial autocorrelation models and the top 2 patch‐based models. Highest total determined the top model

Spatial autocorrelation top models	Early breakup	Late breakup	Total
SASD + ice +refuge + SASD:cos(turning angle)	8	2	10
SASD + ice +refuge + SASD:cos(turning angle) + SASD:ln(step length)	7	2	9
SASD + ice +SASD:cos(turning angle)	6	0	6
No difference	18	25	43

Patch‐based top models	Early breakup	Late breakup	Total
PHAB + refuge +PHAB:cos(turning angle)	13	5	18
PHAB + refuge +PHAB:cos(turning angle) + PHAB:ln(step length)	3	4	7
No difference	23	20	43

For patch‐based analyses, the top iSSA model for the majority of individuals included local PHAB, refuge, and the interaction between PHAB and the cosine of turning angle (Table 1). The AIC values for the top two models were indistinguishable for a number of individuals (raw AIC values in Table S1). Daily sea ice, TE, and PHAB could not be included in any of the same models because of collinearity. Models including PHAB had the lowest AIC of the three variables, and thus PHAB was included in the final model. Three individuals in early breakup and one in late breakup were removed as all used steps and random steps had no variation in PHAB. The top model had beta coefficients that significantly differed from zero for ≥1 of the covariates in 16 of 36 individuals during early breakup (Figure 2c). Nine individuals had a significant negative interaction between PHAB and the cosine of turning angle, reflecting increased changes in the direction of travel with increased PHAB. In late breakup, 8 of 28 individuals had significant beta coefficients for ≥1 of the covariates (Figure 2d). All eight of those individuals had a significant negative interaction reflecting increased changes in the direction of travel with higher PHAB.

In early breakup, the Cox & Snell pseudo‐R‐squared value was higher for the SASD iSSA model in 61.1% of individuals, compared to 27.8% of individuals for the PHAB iSSA. Similarly, in late breakup the pseudo‐R‐squared value was higher for the SASD iSSA in 67.9% of individuals, and PHAB iSSA had higher pseudo‐R‐squared in 25% of individuals. All remaining individuals had the same pseudo‐R‐squared values in both analyses (Table S2).

## 4 DISCUSSION

We used multiple iSSAs to compare two methods of quantifying fragmentation of sea ice habitat used by polar bears. We found that SASD (spatial autocorrelation standard deviation) resulted in better model fits than patch‐based metrics. The iSSA models we tested were better at describing polar bear movement in response to sea ice fragmentation, reflected by the significant interaction between environmental covariate and turning angle, than habitat selection in response to sea ice fragmentation, reflected by the lack of significance in environmental covariates alone.

Using variation in spatial autocorrelation over time to analyze sea ice fragmentation allows for a synthetic, yet complex spatial and temporal visualization of sea ice. SASD allowed for the summarization of habitat over time that would otherwise have to be considered on a daily scale, while still being an effective movement descriptor. Breakup is often defined as a single time span (i.e., McCall et al., 2016; Parks, Derocher, & Lunn, 2006), or even a single date (i.e., Regehr et al., 2007; Sahanatien & Derocher, 2012; Stirling et al., 2004). These definitions over simplify sea ice breakup, which is an important time for polar bears that marks the end of their main seal hunting period (Stirling & McEwan, 1975; Watts & Hansen, 1987), the onset of migration (Cherry et al., 2013), and when their habitat becomes increasingly difficult to traverse. There are multiple marine mammals that use sea ice as primary habitat, all which inhabit regions with an annual ice cycle and experience a period of intense habitat fragmentation when the ice melts (Gagnon & Gough, 2005; Regehr et al., 2007). SASD can be altered by adjusting the size and temporal period of the moving window, thus providing a description of sea ice that is tailored to the spatiotemporal movement scale of the animal. The method can be further altered by using a temporal moving window to explore the effect of SASD at various time periods.

Fragmentation is an important aspect of dynamic sea ice habitat, but it has not been included in habitat modelling for sea ice‐associated marine mammals. Sea ice habitat fragmentation has been considered in a polar bear context for assessing temporal trends in the sea ice season (Sahanatien & Derocher, 2012), but has not been applied to habitat selection. Although aspects of habitat selection by polar bears throughout the year are understood (Mauritzen et al., 2003; McCall et al., 2016; Pilfold, McCall, Derocher, Lunn, & Richardson, 2017), an iSSA enables the exploration of movement alongside habitat selection. Due to its dynamic nature, exploring the fragmented nature of sea ice habitat is complex and adding it into a habitat selection model is computationally intensive. To derive a local patch‐based metric, habitat patches first need to be created from sea ice data, then analyzed to attain the metric outputs; for dynamic habitat such as sea ice, this process must be repeated for each day. As the study area or period increases, working with data of this nature becomes analytically challenging. The creation of the SASD metric allowed us to synthesize daily sea ice fragmentation into a metric that was concise and easier to analyze, while still describing the habitat and the behavior of the animals in the habitat. Spatial autocorrelation has been used in complex habitats where patch‐based metrics would not accurately describe the landscape (Fan & Myint, 2014; Pearson, 2002; Roberts et al., 2000), but the application of this approach to detect wildlife movement in response to complex habitats is novel. We used spatial autocorrelation to include the intricacies of sea ice, as patches tend to create separations in habitat where realistically, they do not exist. Spatial autocorrelation was expanded to become a metric that describes the temporal variability of fragmentation by focusing on the standard deviation over time. In a habitat as dynamic as sea ice, the temporal aspect is inherently important.

Neither SASD nor PHAB showed a quantifiable effect on habitat selection but reveal a connection to movement. Fragmentation covariates described significant habitat selection in few of the individuals, but the benefit of using an iSSA is that we could consider the relationship between our fragmentation metrics and movement. Turning angle is affected by fragmentation, most prominently when fragmentation was quantified using the SASD metric. Extreme changes to sea ice habitat during breakup have an effect on behavior and habitat use, including alterations to migration and feeding behavior (Nilssen, 1995; Pilfold et al., 2017), but the effects of fragmentation within a habitat selection context remain unclear. McCall et al. (2016) quantified polar bear habitat selection during breakup in Hudson Bay using multiple environmental covariates including water depth and distance to various concentrations of ice. This level of habitat selection was not reflected in our analyses as we chose to keep our iSSA simple, focusing on the comparison of fragmentation metrics instead of including numerous environmental covariates.

Although SASD and PHAB model results were comparable, iSSAs using SASD had better model fits for the majority of individuals. In the context of describing movement, SASD is promising because of its ability to describe the relationship between fragmentation and direction of travel. The tendency for SASD to explain movement, rather than habitat selection, could relate to the time frame in which breakup is occurring so that bears are not necessarily selecting for habitat, but rather their ability to move is being affected by the variability of the habitat they encounter while breakup occurs (Cherry et al., 2013; Pilfold et al., 2017). The multiple individuals which showed more changes in directionality in regions of greater SASD could reflect forced changes in direction brought on by habitat changing rapidly during the breakup period. Similarly, models using PHAB, a patch‐based metric, describe movement more effectively than they describe habitat selection during breakup. While this is less intuitive than the relationship between movement and SASD, greater changes in directionality in regions of higher PHAB could be a result of individuals using specific types of ice within the defined optimal habitat for hunting and could explain why a selection signal for PHAB as a whole was not evident. Our defined optimal habitat patches included sea ice cover from 60%–100% so active ice is included in optimal habitat but does not make up the entirety of it. Active ice has greater movement and variation in cover, exhibits more cracks and open water than consolidated ice, and is often related to increased seal availability (Ferguson et al., 2001). The use of active ice could explain changes in direction of travel resulting from both hunting strategy and the dynamic nature of the ice. The grouping of sea ice into patch types could have resulted in the loss of ice variability details which were retained in SASD, thus restricting our ability to interpret PHAB results.

Even though polar bears did not select habitat based on fragmentation, the influence of habitat fragmentation on movement could affect their selection of other habitat components. If fragmentation is a driver of variation in movement, then it is possible the location and severity of fragmented ice could affect selection of other habitat covariates that have been included in other polar bear habitat selection models (i.e., Mauritzen et al., 2003; McCall et al., 2016; Wilson et al., 2016). Including habitat fragmentation in subsequent selection models allows the consideration of ecological trade‐offs between movement toward accessing ideal habitat or resources and the constraints of fragmentation. Variation in spatial autocorrelation of sea ice has the potential to quantify an aspect of sea ice habitat that has been ignored as an inhibitor of selection.

While not the focus of our models, the inclusion of distance to refuge in all top models, and daily sea ice cover in SASD models shows their importance to polar bear movement. Although selection for distance to refuge was not evident in most individuals, breakup is a migratory period for WH polar bears where they generally head toward land (Cherry et al., 2013; McCall et al., 2016). Aspects of local habitat and hunting activity likely affect directed movement toward refuge, resulting in a lack of selection signal. Discontinuous sea ice will reduce an individual's ability to have a straight path toward refuge. Further, directional travel is reduced while hunting because polar bears change direction frequently as they follow scents to locate prey (Ferguson et al., 2001; Smith, 1980). Sea ice cover is also important for polar bear movement because much of their locomotion relies on the presence of ice (Laidre et al., 2018; McCall et al., 2016). The reason for a lack of selection signal for sea ice cover is unclear but could be the result of bears using a range of sea ice cover, as has been in found is previous polar bear habitat selection studies (i.e., Lone et al., 2018; Mauritzen et al., 2003; McCall et al., 2016).

Studies have examined how a changing sea ice season is predicted to affect polar bears (i.e., Castro de la Guardia et al., 2017; Cherry, Derocher, Stirling, & Richardson, 2009; Kovacs et al., 2011; Laidre et al., 2018; Sahanatien & Derocher, 2012), but fine‐scale detail on the effects of sea ice dynamics on individual bears is lacking. Individual fitness is contingent upon the habitat they are exposed to, and success can be variable within a population due to habitat variation (Nilsen, Linnell, & Andersen, 2004; Pettorelli, Gaillard, Duncan, Ouellet, & Van Laere, 2001). Polar bears experience different habitats at an individual level due to the dynamic nature of sea ice. Finding the variation in local‐scale spatial autocorrelation over breakup describes temporal and spatial sea ice patterns which can be applied at an individual scale. Our SASD metric not only provides local descriptions of habitat but also contributes temporal concision that is lacking in patch‐based metrics.

The SASD approach reduced the amount of data needed in the model, but still allowed changes in habitat to be considered in detail. Such an approach may prove useful in identifying important marine habitats for ice‐dependent species or considering broad‐scale habitat change. Polar bears live, travel, mate, and feed on sea ice so alterations to its presence or structure reduce their habitat quality (McCall et al., 2016; Sahanatien & Derocher, 2012). Many seals and walrus require sea ice to be present for the formation of pupping lairs, but they also use ice for hauling out and its presence over shallow waters increases their foraging distribution (Kovacs et al., 2011, Harwood, Smith, Melling, Alikamik, & Kingsley, 2012). For cetaceans with Arctic or subarctic distributions, sea ice influences the availability and distribution of their prey, and most of these species spend the majority of the year in close proximity to ice (Kovacs et al., 2011, Moore, DeMaster, & Dayton, 2000). Seabirds are also affected by the presence of ice, often making use of leads and polynyas for foraging opportunities (Mallory et al., 2010). Variability in sea ice fragmentation affects species that use ice and understanding these dynamics may help uncover important regions for conservation and provide insight on how ice use may change across seasons and years.

## CONFLICT OF INTEREST

None declared.

## AUTHORS’ CONTRIBUTIONS

**Brooke A Biddlecombe:** Conceptualization (lead); Formal analysis (lead); Methodology (lead); Writing‐original draft (lead); Writing‐review & editing (equal). **Erin Bayne:** Conceptualization (supporting); Methodology (supporting); Writing‐review & editing (equal). **Nicholas J. Lunn:** Funding acquisition (equal); Resources (equal); Writing‐review & editing (supporting). **David McGeachy:** Resources (equal); Writing‐review & editing (supporting). **Andrew Edward Derocher:** Conceptualization (supporting); Funding acquisition (equal); Methodology (supporting); Resources (equal); Supervision (lead); Writing‐review & editing (equal). 

## Supporting information

Supplementary Material

## Data Availability

All data are publicly available in Dataverse, https://doi.org/10.7939/DVN/17BG6J
