# Supplementary material for: Comparing sea ice habitat fragmentation metrics using integrated step selection analysis
Source: Ecol Evol. 2020 Apr 12;10(11):4791–800. doi: 10.1002/ece3.6233 (PMC7297736; doi:10.1002/ece3.6233)
Supplement: Supplementary file 1 — Supplementary Material [file ECE3-10-4791-s001.docx]

Comparing sea ice habitat fragmentation metrics using integrated step selection analysis – Supplementary Material

Figure S1. Standard deviation of local Geary’s *c* (quantified using a 5 x 5 pixel moving window) of sea ice cover in each pixel across early breakup, from May 2 to 50% optimal habitat in each year, and late breakup, from 50% optimal habitat in each year to July 23, in the Hudson Bay study area from 2013 – 2018. White zero values represent constant sea ice; grey zero values represent constant open water.

Table S1. Raw AIC values for the top three iSSA spatial autocorrelation models and the top 2 iSSA patch-based models for individual polar bears in early and late breakup.

SASD: Model 1: SASD + ice + refuge + SASD:cos(turning angle)

Model 2: SASD + ice + refuge + SASD:cos(turning angle) + SASD:ln(step length)

Model 3: SASD + ice + SASD:cos(turning angle)

PHAB: Model 1: PHAB + refuge + PHAB:cos(turning angle)

Model 2: PHAB + refuge + PHAB:cos(turning angle) + PHAB:ln(step length)

| ***Early SASD*** | | | | ***Early PHAB*** | | |
| --- | --- | --- | --- | --- | --- | --- |
| **Bear** | **Model 1** | **Model 2** | **Model 3** | **Bear** | **Model 1** | **Model 2** |
| 1 | 343.5 | 344.2 | 348.0 | 1 | 342.4 | 344.4 |
| 2 | 126.0 | 128.1 | 125.5 | 2 | 124.4 | 126.4 |
| 3 | 1850.8 | 1855.9 | 1854.3 | 3 | 1859.3 | 1861.3 |
| 4 | 593.4 | 584.5 | 591.1 | 4 | 565.4 | 567.4 |
| 5 | 252.7 | 252.9 | 250.7 | 5 | 206.3 | 208.3 |
| 6 | 48.3 | 52.9 | 51.3 | 6 | 53.6 | 55.6 |
| 7 | 57.4 | 57.7 | 56.4 | 7 | 53.5 | 55.0 |
| 8 | 711.8 | 712.3 | 709.3 | 8 | 710.1 | 712.1 |
| 9 | 488.9 | 490.9 | 487.3 | 9 | 488.3 | 489.8 |
| 10 | 1313.6 | 1314.2 | 1313.3 | 10 | 1259.4 | 1260.4 |
| 11 | 782.9 | 786.5 | 788.8 | 11 | 795.4 | 795.8 |
| 12 | 89.6 | 95.2 | 92.0 | 12 | 92.0 | 93.3 |
| 13 | 206.6 | 206.7 | 208.4 | 13 | 210.5 | 212.4 |
| 14 | 1811.9 | 1809.0 | 1811.4 | 14 | 1731.0 | 1732.0 |
| 15 | 31.9 | 35.1 | 31.8 | 15 | 31.5 | 33.5 |
| 16 | 1747.8 | 1747.2 | 1747.2 | 16 | 1503.9 | 1484.4 |
| 17 | 366.1 | 367.6 | 367.8 | 17 | 361.5 | 363.3 |
| 18 | 201.0 | 201.9 | 198.7 | 18 | 136.1 | 136.6 |
| 19 | 376.0 | 380.5 | 376.6 | 19 | 355.4 | 357.4 |
| 20 | 886.3 | 887.0 | 884.1 | 20 | 890.1 | 879.1 |
| 21 | 709.2 | 704.3 | 706.2 | 21 | 638.4 | 640.2 |
| 22 | 396.4 | 397.9 | 396.6 | 22 | 397.0 | 399.0 |
| 23 | 808.7 | 801.4 | 806.5 | 23 | 803.8 | 805.7 |
| 24 | 775.1 | 777.7 | 777.1 | 24 | 779.2 | 781.1 |
| 25 | 882.1 | 879.3 | 877.7 | 25 | 870.1 | 872.1 |
| 26 | 218.5 | 220.6 | 216.8 | 26 | 219.4 | 221.3 |
| 27 | 257.2 | 263.4 | 259.8 | 27 | 256.7 | 258.4 |
| 28 | 1417.8 | 1413.3 | 1421.1 | 28 | 1423.2 | 1425.2 |
| 29 | 789.7 | 788.7 | 787.5 | 29 | 693.8 | 695.5 |
| 30 | 901.3 | 903.4 | 899.8 | 30 | 810.8 | 812.7 |
| 31 | 887.6 | 885.5 | 888.8 | 31 | 889.0 | 891.0 |
| 32 | 882.8 | 884.9 | 889.2 | 32 | 891.4 | 860.0 |
| 33 | 3500.6 | 3498.5 | 3501.0 | 33 | 3444.5 | 3446.5 |
| 34 | 2636.7 | 2634.6 | 2645.7 | 34 | 2641.2 | 2643.2 |
| 35 | 1161.2 | 1164.4 | 1162.6 | 35 | 1167.7 | 1169.6 |
| 36 | 1004.3 | 1001.0 | 998.5 | 36 | 1006.0 | 1007.6 |
| 37 | 1866.1 | 1869.8 | 1868.1 | 37 | 1800.0 | 1801.9 |
| 38 | 1687.5 | 1687.4 | 1685.1 | 38 | 1683.2 | 1685.0 |
| 39 | 1394.0 | 1394.3 | 1405.0 | 39 | 1389.9 | 1391.9 |
| ***Late SASD*** | | | | ***Late PHAB*** | | |
| **Bear** | **Model 1** | **Model 2** | **Model 3** | **Bear** | **Model 1** | **Model 2** |
| 1 | 8.0 | 10.0 | 7.3 | 1 | 6.0 | 8.0 |
| 2 | 48.3 | 49.5 | 50.5 | 2 | 48.9 | 50.8 |
| 3 | 433.9 | 434.3 | 432.0 | 3 | 440.5 | 442.4 |
| 4 | 448.8 | 444.1 | 447.0 | 4 | 442.3 | 441.8 |
| 5 | 31.7 | 33.3 | 29.8 | 5 | 33.5 | 33.4 |
| 6 | 53.6 | 55.6 | 52.5 | 6 | 51.8 | 53.3 |
| 7 | 415.9 | 417.9 | 414.2 | 7 | 418.0 | 419.2 |
| 8 | 258.4 | 260.3 | 257.1 | 8 | 260.3 | 262.3 |
| 9 | 50.7 | 52.1 | 49.3 | 9 | 14.8 | 14.8 |
| 10 | 340.6 | 342.6 | 338.8 | 10 | 333.6 | 335.6 |
| 11 | 256.5 | 258.5 | 260.1 | 11 | 224.9 | 226.8 |
| 12 | 86.6 | 85.7 | 85.5 | 12 | 83.1 | 84.7 |
| 13 | 315.9 | 312.1 | 314.0 | 13 | 295.3 | 289.8 |
| 14 | 59.5 | 61.1 | 57.7 | 14 | 57.3 | 58.9 |
| 15 | 38.1 | 39.3 | 37.5 | 15 | 77.7 | 77.9 |
| 16 | 337.6 | 339.6 | 339.9 | 16 | 343.4 | 344.5 |
| 17 | 246.9 | 244.0 | 245.0 | 17 | 368.0 | 348.2 |
| 18 | 467.2 | 469.0 | 466.9 | 18 | 470.5 | 469.7 |
| 19 | 42.4 | 30.5 | 46.7 | 19 | 45.3 | 39.7 |
| 20 | 177.1 | 178.3 | 175.2 | 20 | 183.9 | 185.8 |
| 21 | 331.8 | 333.7 | 330.0 | 21 | 358.1 | 339.2 |
| 22 | 103.6 | 105.4 | 101.7 | 22 | 101.7 | 103.7 |
| 23 | 936.5 | 938.5 | 936.6 | 23 | 932.2 | 931.9 |
| 24 | 1640.1 | 1642.1 | 1638.7 | 24 | 1641.2 | 1643.0 |
| 25 | 83.6 | 85.5 | 81.7 | 25 | 73.3 | 72.7 |
| 26 | 116.5 | 118.5 | 114.8 | 26 | 118.0 | 117.9 |
| 27 | 188.9 | 188.6 | 186.9 | 27 | 188.2 | 189.6 |
| 28 | 935.3 | 936.9 | 933.4 | 28 | 979.2 | 974.8 |
| 29 | 592.4 | 594.0 | 593.1 | 29 | 589.2 | 590.4 |

Table S2. Cox & Snell pseudo R-squared values from integrated step selection analyses comparing two methods of quantifying habitat fragmentation: standard deviation of Geary’s *c* (SASD) and local percentage of optimal habitat (PHAB) using locations from adult female polar bears in Hudson Bay from 2013-2018. Maximum possible pseudo R-squared value for all models was 0.353. Individuals removed from analyses denoted as n/a.

| Early Breakup | | | Late Breakup | | |
| --- | --- | --- | --- | --- | --- |
| **Individual Bear** | **SASD pseudo**  **R-squared** | **PHAB pseudo**  **R-squared** | **Individual Bear** | **SASD pseudo**  **R-squared** | **PHAB pseudo**  **R-squared** |
| 1 | 0.012 | 0.011 | 1 | 0.353 | 0.353 |
| 2 | 0.007 | 0.005 | 2 | 0.067 | 0.045 |
| 3 | 0.003 | 0.001 | 3 | 0.015 | 0.007 |
| 4 | 0.01 | 0.029 | 4 | 0.151 | 0.153 |
| 5 | 0.008 | 0.005 | 5 | 0.075 | 0.02 |
| 6 | 0.067 | 0.003 | 6 | 0.021 | 0.02 |
| 7 | 0.027 | n/a | 7 | 0.01 | 0.005 |
| 8 | 0.001 | 0.001 | 8 | 0.029 | 0.023 |
| 9 | 0.003 | 0.001 | 9 | 0.075 | n/a |
| 10 | 0.003 | 0.009 | 10 | 0.009 | 0.004 |
| 11 | 0.014 | 0.009 | 11 | 0.032 | 0.008 |
| 12 | 0.023 | 0.002 | 12 | 0.015 | 0.017 |
| 13 | 0.025 | 0.013 | 13 | 0.01 | 0.01 |
| 14 | 0.002 | 0.004 | 14 | 0.01 | 0.012 |
| 15 | 0.071 | 0.049 | 15 | 0.108 | 0.002 |
| 16 | 0.009 | 0.01 | 16 | 0.014 | 0.004 |
| 17 | 0.008 | 0.011 | 17 | 0.018 | 0.003 |
| 18 | 0.006 | 0.015 | 18 | 0.01 | 0.005 |
| 19 | 0.007 | 0.004 | 19 | 0.084 | 0.038 |
| 20 | 0.003 | 0.002 | 20 | 0.031 | 0.01 |
| 21 | 0.001 | 0.001 | 21 | 0.015 | 0.003 |
| 22 | 0.005 | 0.002 | 22 | 0.022 | 0.021 |
| 23 | 0.003 | 0.004 | 23 | 0.014 | 0.015 |
| 24 | 0.005 | 0.002 | 24 | 0.002 | 0.001 |
| 25 | 0.002 | 0.002 | 25 | 0.006 | 0.052 |
| 26 | 0.037 | n/a | 26 | 0.007 | 0.012 |
| 27 | 0.016 | 0.006 | 27 | 0.014 | 0.011 |
| 28 | 0.007 | 0.005 | 28 | 0.038 | 0.018 |
| 29 | 0.009 | 0.008 | 29 | 0.004 | 0.005 |
| 30 | 0.005 | 0.013 |  | | |
| 31 | 0.006 | 0.004 |  |  |  |
| 32 | 0.004 | 0.006 |  |  |  |
| 33 | 0.001 | 0.001 |  |  |  |
| 34 | 0.003 | 0.002 |  |  |  |
| 35 | 0.005 | 0.001 |  |  |  |
| 36 | 0.003 | 0.001 |  |  |  |
| 37 | 0.001 | 0.004 |  |  |  |
| 38 | 0.005 | 0 |  |  |  |
| 39 | 0.004 | n/a |  |  |  |
